# Characterization of Posttranslationally Modified Multidrug Efflux Pumps Reveals an Unexpected Link between Glycosylation and Antimicrobial Resistance

**DOI:** 10.1128/mBio.02604-20

**Published:** 2020-11-17

**Authors:** Sherif Abouelhadid, John Raynes, Tam Bui, Jon Cuccui, Brendan W. Wren

**Affiliations:** a Department of Pathogen Biology, London School of Hygiene and Tropical Medicine, London, United Kingdom; b Biomolecular Spectroscopy Centre, King’s College London, Hodgkin, United Kingdom; Department of Veterinary Medicine

**Keywords:** multidrug efflux pump, *N*-linked glycans, glycosylation

## Abstract

The substantial rise in multidrug-resistant bacterial infections is a current global imperative. Cumulative efforts to characterize antimicrobial resistance in bacteria has demonstrated the spread of six families of multidrug efflux pumps, of which resistance-nodulation-cell division (RND) is the major mechanism of multidrug resistance in Gram-negative bacteria. RND is composed of a tripartite protein assembly and confers resistance to a range of unrelated compounds. In the major enteric pathogen Campylobacter jejuni, the three protein components of RND are posttranslationally modified with *N-*linked glycans. The direct role of *N-*linked glycans in C. jejuni and other bacteria has long been elusive. Here, we present the first detailed account of the role of *N-*linked glycans and the link between *N-*glycosylation and antimicrobial resistance in C. jejuni. We demonstrate the multifunctional role of *N-*linked glycans in enhancing protein thermostability, stabilizing protein complexes and the promotion of protein-protein interaction, thus mediating antimicrobial resistance via enhancing multidrug efflux pump activity. This affirms that glycosylation is critical for multidrug efflux pump assembly. We present a generalized strategy that could be used to investigate general glycosylation system in *Campylobacter* genus and a potential target to develop antimicrobials against multidrug-resistant pathogens.

## Abstract (precis)

Nearly all bacterial species have at least a single glycosylation system, but the direct effects of these posttranslational protein modifications are unresolved. Glycoproteome-wide analysis of several bacterial pathogens has revealed general glycan modifications of virulence factors and protein assemblies. Using Campylobacter jejuni as a model organism, we have studied the role of general *N-*linked glycans in the multidrug efflux pump commonly found in Gram-negative bacteria. We show, for the first time, the direct link between *N*-linked glycans and multidrug efflux pump activity. At the protein level, we demonstrate that *N*-linked glycans play a role in enhancing protein thermostability and mediating the assembly of the multidrug efflux pump to promote antimicrobial resistance, highlighting the importance of this posttranslational modification in bacterial physiology. Similar roles for glycans are expected to be found in other Gram-negative pathogens that possess general protein glycosylation systems.

## INTRODUCTION

The emergence of antimicrobial resistance in bacterial pathogens is considered a public health crisis causing serious life-threating infections worldwide. Multidrug-resistant Gram-negative bacteria are estimated to attribute to about 80% of all severe infections observed clinically (1). Seeking new routes to develop new antimicrobials against Gram-negative bacteria is a current global imperative. The mechanisms by which bacteria confer antimicrobial resistance have been widely investigated. Among the bacterial repertoire that mediate antimicrobial resistance, the resistance-nodulation-cell division (RND) family is considered a major key player in the occurrence of such a phenotype in Gram-negative bacteria. RND typically consists of an inner membrane protein, a periplasmic fusion protein and an outer membrane channel (2). This tripartite assembly confers resistance to structurally unrelated compounds such as bile salts, heavy metals, and antimicrobials, thus promoting bacterial survival not only in the mammalian host but also a range of environmental niches (1). Mutational studies in RND have demonstrated a significant reduction in bacterial competitiveness and virulence in bacterial pathogens such as Salmonella enterica serovar Typhimurium (3), Francisella tularensis (4), Vibrio cholerae (5), Neisseria gonorrhoeae (6), and Campylobacter jejuni (7).

**FIG 1:**
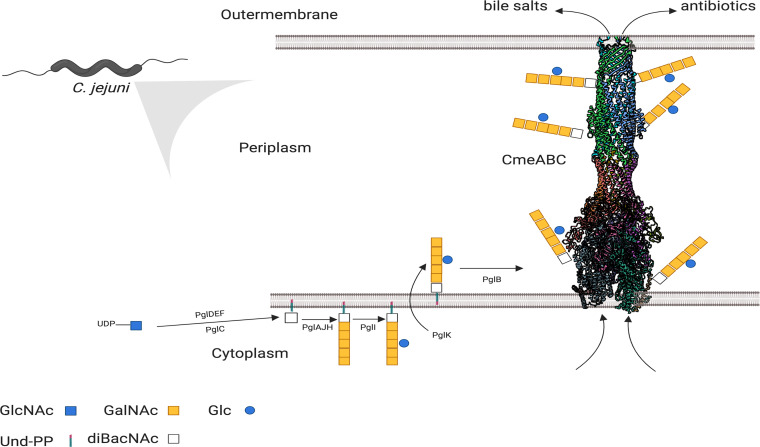
Schematic diagram of *N*-linked glycosylation pathway in C. jejuni and model for the role of glycosylation in the functioning of multidrug efflux pumps. Each protein component of CmeABC carries two glycosylation sites whereby PglB catalyzes the transfer of the *N*-linked glycans to CmeABC. CmeA is glycosylated at positions ^121^DFNRS^125^ and ^271^DNNNS^275^, CmeB is glycosylated at position ^634^DRNVS^648^ and theoretically at ^663^DRNAS^667^, and CmeC is glycosylated at position ^47^ETNSS^51^ and theoretically at ^30^EANYS^34^.

The bacterium C. jejuni is a major cause of gastroenteritis worldwide. Studies have estimated the disease burden of C. jejuni to be associated with 7.5 million disability-adjusted life years, exceeding *Shigella* (7.1 million) and enterotoxigenic Escherichia coli (6.9 million) (8). Disease presentation varies between self-limiting illness manifested by diarrhea, fever and malaise, to autoimmune conditions such as the Guillain-Barre and Miller-Fischer syndromes (9). The foodborne pathogen C. jejuni is transmitted to humans mainly via the consumption and handling of undercooked poultry meat, dairy products, water, and unpasteurized milk (10). Due to its prevalence in the intestines of food-producing animals and the exposure to antibiotics either used in animal production and/or human medications, C. jejuni has evolved several antimicrobial resistance mechanisms (11). As a result, both the Centre for Disease Control and Prevention (CDC) and the World Health Organization have recently listed multidrug-resistant C. jejuni as a serious antibiotic resistance threat (12, 13). In C. jejuni, the RND protein assembly CmeABC is considered the predominant major multidrug efflux system. CmeABC is composed of a tripartite molecular assembly of glycoproteins: CmeB, an inner membrane multidrug transport protein; CmeA, a periplasmic fusion protein, and CmeC, an outer membrane-associated channel (14, 15) (Fig. 1). The complex has been shown to be important for C. jejuni to colonize chickens, as well as being responsible for intrinsic and acquired multidrug resistance in the bacterium (7). This efflux system is tightly regulated by the transcriptional repressor CmeR and/or CosR, both of which bind to the promoter region of the *cmeABC* operon (16, 17). Transcriptional studies have demonstrated that mutations in CmeR or alteration in promoter sequence lead to an overexpression of CmeABC (17, 18).

Glycoproteome analysis of C. jejuni has revealed that CmeA, CmeB, and CmeC are decorated by an *N-*linked heptasaccharide glycan (GalNAc-α1,4-GalNAc-α1,4-[Glc-β-1,3]GalNAc-α1,4-GalNAc-α1,4-GalNAc-α1,3-diNAcBac, where GalNAc is *N-*acetylgalactosamine, Glc is glucose, and diNAcBac is 2,4-diacetamido-2,4,6-trideoxyglucopyronose) attached to the asparagine residue in the acceptor sequon D/E-X_1_-N-X_2_-S/T with X_1_ and X_2_ as any amino acid except proline (19). Genetic mutational studies and glycomic analysis have already demonstrated that the *N-*oligosaccharyltransferase PglB is the key enzyme that catalyzes *N-*glycosylation of, at least, 53 proteins in C. jejuni (19, 20). While the direct role of *N-*linked glycan remains to be elucidated, disruption of the *N*-glycosylation pathway results in pleiotropic effects such as decreased chicken colonization, reduced adherence to intestinal cells, and impaired bacterial competence (21–23). Previously, we have demonstrated that disrupting *pglB* impaired the efflux activity of CmeABC, resulting in significantly higher ethidium bromide accumulation compared to the wild type (24). Ablation of glycosylation on CmeABC was also shown to reduce resistance to four different antibiotic classes (24). In the present study, we show that the loss of the *N-*linked glycans in CmeABC is the sole reason for the multidrug efflux pump impairment phenotype and not a pleiotropic effect caused by the disruption of the *N-*oligosaccharyltransferase *pglB*. We also unravel the intrinsic role of the posttranslational modification of CmeABC in (i) modulating global protein structure, (ii) enhancing glycosylated CmeA (g2CmeA) thermostability, and (iii) significantly slowing the unfolding rate of g2CmeA. Finally, we evaluate the extrinsic role of *N-*linked glycans in the molecular assembly of CmeABC to discern the difference in the binding kinetics of CmeA variants to CmeC. The present study highlights the multifunctional role of *N-*linked glycans in enhancing protein thermostability, stabilizing protein complexes, and the promotion of protein-protein interaction to mediate antimicrobial resistance via enhancing multidrug efflux pump activity. We also present a model *N*-linked glycosylation system with a tractable phenotype that can be used to facilitate the study of glycans in evolution, function, and diversity.

## RESULTS

### *N*-linked glycans affect multidrug efflux pump efficiency.

**FIG 2:**
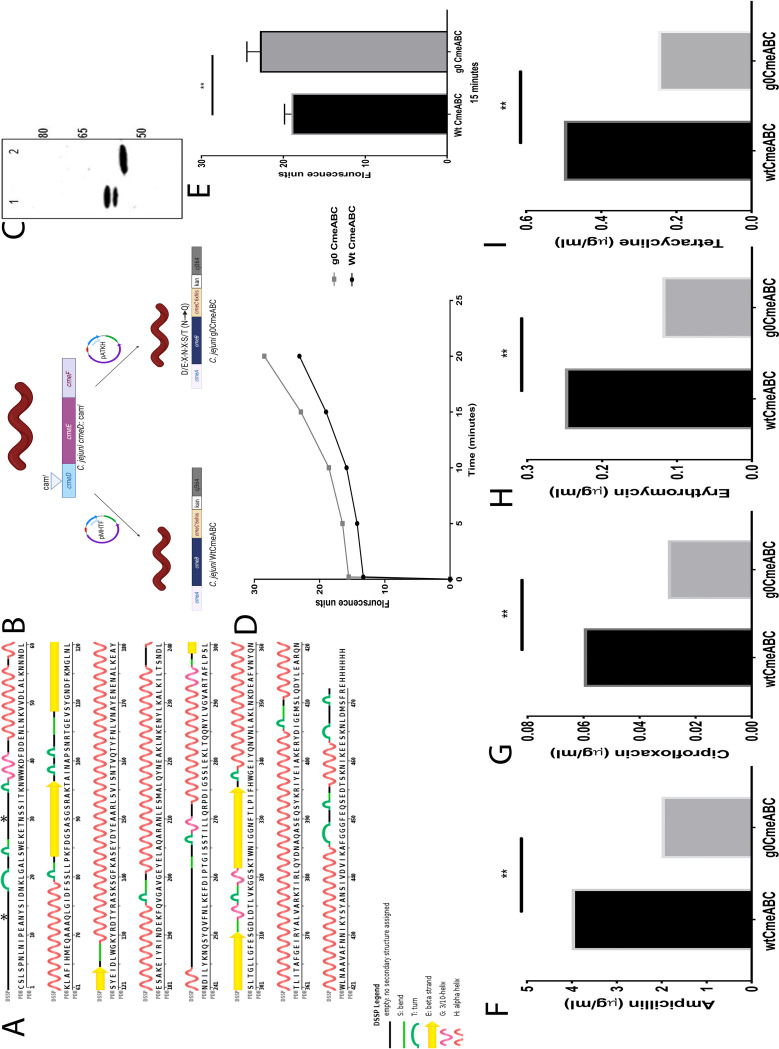
Functional studies and effect of glycosylation on WTCmeABC and g0CmeABC. (A) DSSP analysis of CmeC (PDB 4TM4). Glycosylation sites ^47^ETNSS^51^ (reported) and ^0^EANYS^34^ are located in a flexible loop. Glycosylation sites in CmeC are denoted by asterisk. (B) Construction of C. jejuni variants. Inactivation of *cmeD* was achieved by introducing chloramphenicol cassette in the middle of the gene. This strain was later used as a parent strain to construct WTCmeABC and g0CmeABC, whereby glycosylation of CmeABC was disabled by introducing N→Q amino acid alteration to asparagine in D/E-X-N-X-S/T (X = any amino acid other than proline). (C) Western blot detection of CmeC. WTCmeABC and g0CmeABC strains were grown overnight in brucella broth media, and cells were pelleted and incubated with 2% sodium dodecyl sulfate and sodium Sarkosyl for 2 h at room temperature. The cell debris was then pelleted by centrifugation, and supernatants were mixed 1:1 with Laemmli loading buffer supplemented with dithiothreitol. Proteins were then separated by SDS-PAGE, followed by electroblotting to a polyvinylidene difluoride membrane. His_6_-tagged CmeC was probed by primary anti-His_6_ mouse antibody and visualized by using a LI-COR Odyssey apparatus. (D) Ethidium bromide accumulation test in C. jejuni strains. Brucella broth (30 ml) was separately inoculated with an overnight culture of C. jejuni WTCmeABC (black) and C. jejuni g0CmeABC (gray) to an OD_600_ of 0.1. Cells were grown until reaching an OD_600_ of 0.4 to 0.5 and then spun down, washed, and resuspended to an OD_600_ of 0.2 in 10 mM sodium phosphate buffer (pH 7). The cells were incubated in a VAIN apparatus for 15 min at 37°C. Ethidium bromide was then added to final concentration of 0.2 mg/ml. (D) The fluorescence was read at excitation and emission for 20 min at 37°C. (E) Eithidum bromide accumulation in C. jejuni strains at 15 min. (F to I) MICs of C. jejuni WTCmeABC and C. jejuni g0CmeABC. The MIC was read directly from the strip at the point where the zone of inhibition of bacterial growth intersected with the antibiotic concentration on the strip. The data represent the means of three biological replicates, with two technical replicates for each. Significance was calculated using Mann-Whitney test (**, *P* < 0.01).

Scrutiny of the C. jejuni NCTC11168 genome reveals the presence of several predicted efflux transporters, which appear conserved in the species (25). Genetic and biochemical testing has demonstrated that *cmeABC* is located in an operon and encodes the predominant multidrug efflux pump in C. jejuni (14). CmeABC plays a central role in extruding structurally nonrelated compounds such as antimicrobials, bile salts, dyes, and heavy metals (7, 14). Glycoproteomic analysis of C. jejuni has demonstrated that CmeA is glycosylated at positions ^121^DFNRS^125^ and ^271^DNNNS^275^; CmeB is glycosylated at position ^634^DRNVS^648^, and CmeC is glycosylated at position ^47^ETNSS^51^ (19). Interestingly, our bioinformatic analysis of CmeB and CmeC has revealed two additional glycosylation sites, ^663^DRNAS^667^ and ^30^EANYS^34^, respectively, located in nonstructural regions that are potentially accessible by PglB, hence more likely to be glycosylated (Fig. 2A; see also Fig. S1 in the supplemental material). Previously, we showed that the multidrug efflux pump is impaired in a glycosylation deficient C. jejuni, resulting in a significant increase in ethidium bromide accumulation and a reduction in antibiotic resistance compared to the wild-type strain (24). We hypothesize that this deficiency may be due to assembly destabilization as a consequence of the glycosylation removal. To address this question, we sought to study the major multidrug efflux pump of C. jejuni, in a glycosylation-null CmeABC complex.

CmeABC has been reported to function interactively with CmeDEF (26), a secondary multidrug efflux pump complex in C. jejuni. Mutagenesis studies have demonstrated that CmeDEF confers intrinsic resistance to antimicrobials and toxic compounds in C. jejuni. We designed our experiments to be conducted in a C. jejuni *cmeD*::*cat*^r^ background to avoid a potential interaction between CmeDEF and CmeABC which might mask the functional role of *N-*linked glycans (26). The secondary multidrug efflux pump, CmeDEF, was disrupted by the introduction of chloramphenicol resistance cassette within the gene encoding outer membrane efflux protein, *cmeD.* This parent strain was then used to construct a C. jejuni wild-type CmeABC (WTCmeABC) strain and a C. jejuni glycosylation altered strain, g0CmeABC, whereby the asparagine residue (N) in each (both reported and predicted) glycosylation sequon (D/E-X_1_-N-X_2_-S/T, where X_1_ and X_2_ are any amino acids other than proline) was mutated to glutamine (Q) (Fig. 2B). This is one of the most conserved amino acid alterations (27), since N and Q differ only by a single methylene group; we also added a His_6_ tag at the C terminus of CmeC. Immunoblot analysis demonstrated difference in migration between His-tagged WTCmeC (lane 1) compared to its glycosylation-deficient counterpart (lane 2) (Fig. 2C). Notably, WTCmeC showed two bands migrating more slowly than g0CmeC. This result shows the potential occupation of an additional glycosylation site at CmeC with the *N-*linked glycans. Both of the bands, in WTCmeC, reacted positively when probed by SBA lectin, which preferentially binds to oligosaccharides with *N-*acetylgalactosamine as terminal sugar (see Fig. S2A to C in the supplemental material).

10.1128/mBio.02604-20.1 FIG S1 DSSP analysis of CmeB (PDB 5LQ3). Glycosylation sites ^634^DRNVS^648^ and theoretically at ^663^DRNAS^667^ are denoted by asterisks. Download FIG S1, JPG file, 0.1 MB. Copyright © 2020 Abouelhadid et al. 2020 Abouelhadid et al. This content is distributed under the terms of the Creative Commons Attribution 4.0 International license.

10.1128/mBio.02604-20.2 FIG S2 Western blot detection of CmeC variants, lane 1, WTCmeC; lane 2, g0CmeC. Proteins separated by SDS-PAGE electroblotted onto polyvinylidene difluoride membranes, probed by anti-His_6_ and SBA lectin, and visualized using a LI-COR Odyssey. Anti-His_6_ signal is indicated in red (a), SBA lectin signal is indicated in green (b), and both images are superimposed (c). Download FIG S2, JPG file, 0.02 MB. Copyright © 2020 Abouelhadid et al. 2020 Abouelhadid et al. This content is distributed under the terms of the Creative Commons Attribution 4.0 International license.

Next, we examined the role of *N-*linked glycans in the CmeABC molecular assembly, and we assessed the efficiency of the multidrug efflux pump using an ethidium bromide accumulation assay. Ethidium bromide accumulation was 22% higher in g0CmeABC than in WTCmeABC. This difference was consistent at 5, 10, and 15 min, indicating impairment in the extrusion of ethidium bromide from g0CmeABC (Fig. 2D). To confirm this finding, Etest antibiotic strips were used to calculate the MICs of four nonstructurally related antibiotics that have different mechanisms of actions. According to the U.S. Centers for Disease Control and Prevention (CDC), azithromycin (an analogue of erythromycin) is used for the treatment of C. jejuni. We tested the effect of *N-*glycans on erythromycin as a clinically relevant antibiotic. In comparison to WTCmeABC, a 100% increase in antibiotic susceptibility in the four different antibiotics tested was noticed in g0CmeABC (Fig. 2F to I). These results demonstrate a consistent decrease in multidrug efflux pump efficiency when glycosylation is no longer available, thus confirming the phenotype seen in the ethidium bromide accumulation test. The results indicate that *N-*linked glycans play a role in enabling the multidrug efflux pump to work efficiently in the C. jejuni cell.

### Generation of fully glycosylated CmeA in glycocompetent *E. coli*.

To further investigate the role of *N-*linked glycans on modulating the function of CmeABC, we heterologously expressed nonglycosylated CmeA (denoted as g0CmeA) and glycosylated CmeA (denoted as g2CmeA) in E. coli.

**FIG 3:**
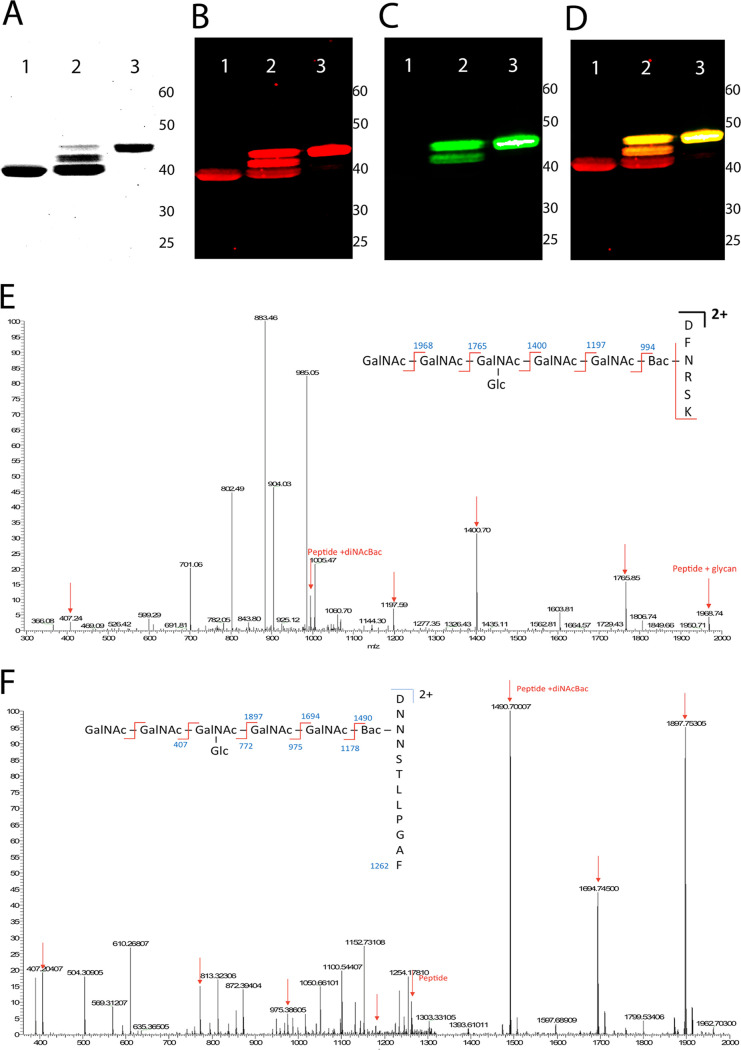
Generation and mass spectrometry analysis of fully glycosylated CmeA. CmeA was purified using IMAC, followed by concentration and buffer exchange using Amicon Ultra 0.5-ml centrifugal filter units. Proteins were then separated by SDS-PAGE and visualized by Coomassie blue staining (A) or electroblotted to a polyvinylidene difluoride membrane (B). CmeA-His_6_ was probed using anti-His_6_ mouse antibody (B) or SBA lectin (C) and then visualized by a Li-COR Odyssey, which allowed two-colors immunoblot (D). Lane 1, g0CmeA from E. coli DH10B; lane 2, gCmeA from SDB1 carrying pACYC(*pgl*) and pWA2; lane 3, fully glycosylated CmeA was produced in E. coli SDB1 carrying pEXT21Cj*pglB*, pACYC(*pgl*), and pWA2. (E and F) Mass spectrometry analysis of glycopeptides from CmeA. Spectra were produced by fragmentation of the glycan structure attached to two glycosylation sites in g2CmeA digested by trypsin (DFNRS) (E) or chymotrypsin (DNNNS) (F). Peaks indicative of fragmentation of the *N-*glycans are highlighted by red arrows, whereas peptide *m/z* values and peptides with diNAcBac are indicated in blue.

This strategy allowed us to investigate whether the phenotype observed in WTCmeABC and g0CmeABC strains is solely due to abrogation of glycosylation and not a secondary effect in C. jejuni. Previous studies have shown that UDP-*N-*acetylglucosamine–undecaprenyl-phosphate *N*-acetylglucosaminephosphotransferase (WecA) could interfere with the biosynthesis of heterologous expression of oligosaccharides built on the undecaprenyl-phosphate lipid anchor, thus replacing the reducing end sugar of the oligosaccharide with an incorrect sugar, GlcNAc (28). To circumvent this problem and ascertain that g2CmeA is glycosylated with the native C. jejuni *N*-linked glycan, we used a glycocompetent E. coli *wecA* strain, SDB1 (29). The heterologous expression of an acceptor protein with protein glycosylation locus (*pgl*) usually yields a mix population of glycosylated and nonglycosylated protein variants, indicating a suboptimal glycosylation process (28) (Fig. 3A, lane 2). We observed that *pglB* expression from pACYC(*pgl*) was insufficient to attain CmeA full glycosylation. To overcome this bottleneck, we sought to boost PglB expression by introducing pEXT21Cj*pglB* to E. coli SDB1 expressing a soluble periplasmic CmeA and *N*-linked glycan biosynthetic pathway. Efficient glycosylation was achieved by constitutively expressing CmeA and *N*-linked glycosylation pathway along with IPTG (isopropyl-β-d-thiogalactopyranoside)-inducible PglB from the pEXT21Cj*pglB* backbone (Fig. 3A, lane 3). Thus, the yield of g2CmeA was 0.2 mg/liter compared to 0.6 mg/liter for g0CmeA.

We then used mass spectrometry analysis to precisely define *N-*linked decorating CmeA. In-gel digestion was carried out, and the resulting peptides were analyzed by liquid chromatography-tandem mass spectrometry (LC-MS/MS). CmeA was identified after the raw data were searched at a stringency threshold of a 1% false discovery rate (FDR) for protein and a minimum of one peptide per protein as determined by Mascot and Sequest in the Proteome Discoverer method. Further data analysis was performed with the addition of the accurate mass of the heptasaccharide glycan (C_56_H_91_N_7_O_34_) to the modification list in the database search method. This mass was calculated (with the inclusion of the diNAcBac mass) at 1,405.7293 Da, allowing for addition to asparagine residues. Data analysis indicated that two doubly charged peptides were identified carrying this glycan modification: DFNRSK and DNNNSTLLPGAF. Fragmentation of glycosylated DFNRSK showed the loss of individual sugar residues of the *N-*linked glycan and yielded a peptide mass-to-charge (*m/z* 993.93), which—potentially—consists of diNAcBac (*m/z* 228) attached to DFNRSK (*m/z* 765.82) (Fig. 3E). Fragmentation of the second glycosylated peptide, DNNNSTLLPGAF, showed a peak of *m/z* 1,490, indicating an extra 228 Da attached to the peptide. The loss of the 228 Da yielded a peak of *m/z* 1,262, which corresponds to the peptide mass only (Fig. 3D). Since the loss of 228 Da is consistent with the identity of diNAcBac residue and there was no indication of a direct attachment of a HexNAc residue to any of the glycopeptides, our mass spectrometry analysis confirms the glycan identity attached to CmeA to be the native C. jejuni heptasaccharide, built on the correct reducing end sugar, diNAcBac.

### Glycosylation modulates protein global structure.

**FIG 4:**
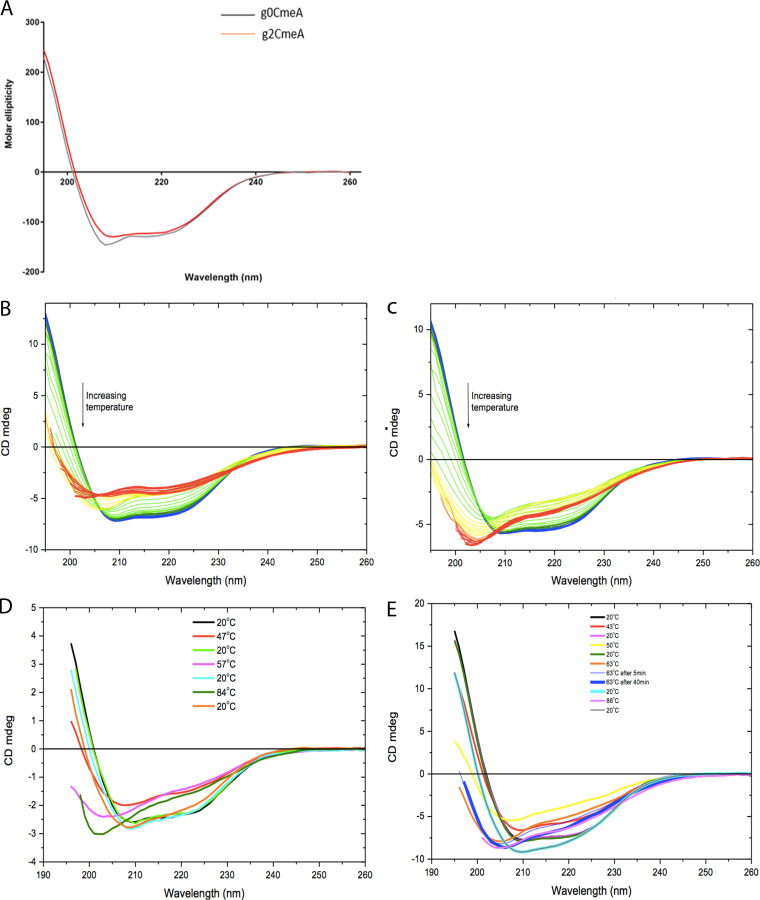
CD spectra of CmeA variants in 10 mM sodium phosphate, 75 mM sodium chloride, and 10% glycerol (pH 8.0). (A) Far-UV CD spectra were collected for g0CmeA (0.124 mg/ml) and g2CmeA (0.174 mg/ml) variants using a 0.5-mm rectangular cell pathlength. The molar ellipticity was calculated and corrected for protein concentrations. Each point represents an average of collected reads made every 25 μs for 1.5 ms; the data represent the averages of three biological repeats. Thermal melts of CmeA variants for far-UV CD spectra were collected for g0CmeA (0.124 mg/ml) and g2CmeA (0.174 mg/ml) variants using a 0.5-mm rectangular cell pathlength. CD mdeg values were recorded as a function of temperature from blue (6°C) to red (94°C) for g0CmeA (B) and g2CmeA (C). Each color in between was obtained at rate 1°C/min with a 2°C stepwise increase. The reversibility of thermal unfolding was achieved recorded at 20°C, raised to *T_m_*, and recooled to 20°C sequentially. The CD spectra were collected for 5 min at each temperature interval for g0CmeA (D) and g2CmeA (E). The CD spectra of g2CmeA stabilized after 30 min at *T_m_*_3_, indicating a more resilient behavior thermal unfolding process.

To discern the role that *N-*linked glycans play in modulating the biophysical properties of CmeABC, we used circular dichroism (CD) spectroscopy. This allowed us to monitor the secondary structure, as well as the conformational changes upon thermal denaturation of CmeA variants. Data were collected every 25 μs for 1.5 ms. Far-UV spectra for both g0CmeA and g2CmeA in 10 mM sodium phosphate, 75 mM sodium chloride, and 10% glycerol buffer (pH 8) were collected at 20°C. The CD spectrum indicated the two characteristic negative minima at 208 and 222 nm of α-helical conformations and a positive maximum at 196 nm, suggesting the presence of β-sheet conformation (Fig. 4A). Superimposed CD spectra of the g0CmeA spectrum, shown in black, were slightly red shifted toward the β sheet. The CD spectra of both proteins were then analyzed by BESTSEL (30) for secondary structure content. Table 1 shows the mean of secondary structure content of g0CmeA and g2CmeA from three replicates calculated at room temperature. Subtle differences were observed among the protein global structures of CmeA variants. On one hand, *N-*linked glycans attachment to g2CmeA seems to favor the protein to adopt a more α-helical conformation, 25.4% g2CmeA compared to 23.2% g0CmeA. On the other hand, glycosylation seems to be affecting β-sheet conformations in g2CmeA, whereby the g0CmeA content was slightly higher than that for g2CmeA, 29 and 28.2%, respectively. Our results show that *N-*linked glycans confer distinct changes in protein global conformation.

**TABLE 1:** Secondary structure calculation of g0CmeA and g2CmeA variants^*a*^

Variant	Content (%)			
	α-Helix	β-Sheet	Turn	Others
g0CmeA	23.2	29	12.1	35.5
g2CmeA	25.4	28.2	10.9	35.3

a CD units were converted to delta epsilon units and loaded into the BESTSEL server. Although the conformations of both proteins are structurally similar, there is a subtle shift in the alpha-helix and beta-sheet ratios between the two variants.

### Glycans enhance protein thermostability.

The intrinsic role of *N-*linked glycans in stabilizing CmeA was investigated by analyzing the CD spectra recorded for g0CmeA and g2CmeA at elevated temperatures. The multiwavelength melting profiles monitored at 260 to 195 nm were recorded during the heating of g0CmeA and g2CmeA from 6 to 94°C at a 1°C/min rate with a 2°C step size. More than one isodichroic point was observed in the far-UV CD spectra (Fig. 4B and C), indicating that the unfolding transition is a multistep process. CD derivative spectra were used to calculate the melting temperatures (*T_m_*s) of both g0CmeA and g2CmeA. A reduction of the CD spectrum intensity was observed upon incremental rises in temperature. Melting curves measured for independent three biological replicates of CmeA variants show two transition phases for *T_m_*_1_ = 45.5°C ± 1.6 and *T_m_*_2_ = 58.4°C ± 0.7 for CmeA, but three transition phases for *T_m_*_1_ = 43.2°C ± 0.6, *T_m_*_2_ = 49.1°C ± 0.2, and *T_m_*_3_ = 62.5°C ± 0.6 for g2CmeA. This shift in final melting temperature suggests that glycans thermally stabilize g2CmeA at elevated temperature (Fig. 4C).

10.1128/mBio.02604-20.3 FIG S3 Quantification of CmeC variants. Immunoblot images of CmeC variants were analyzed by using Image. Download FIG S3, JPG file, 0.02 MB. Copyright © 2020 Abouelhadid et al. 2020 Abouelhadid et al. This content is distributed under the terms of the Creative Commons Attribution 4.0 International license.

To confirm the previous findings, we examined the conformational folding reversibility and unfolding rate for both g0CmeA and g2CmeA. The assay is based on successive cycles wherein CmeA variants were cooled at 20°C, heated up to the corresponding *T_m_* for 5 min, and then cooled again to 20°C. To assess conformational folding reversibility, CD spectra that were recorded at 20°C, before and after increasing the temperature to the corresponding *T_m_*, were compared. The CD spectra of CmeA were superimposable before and after the first cycles of heating (*T_m_*_1_) but not after heating at *T_m_*_2_, while the g2CmeA CD spectra were superimposable before and after the first two cycles (*T_m_*_1_ and *T_m_*_2_) but not after heating at *T_m_*_3_. This observation might indicate protein aggregation in CmeA variants due to conformational changes (Fig. 4D and E). The unfolding rate was evaluated according to changes in the CD spectra with respect to time at *T_m_*_2_ for CmeA and *T_m_*_3_ for g2CmeA. A significant reduction in the CD spectra intensity was observed when g0CmeA and g2CmeA were heated at their corresponding *T_m_*_2_ and *T_m_*_3_, respectively. The unfolding of g0CmeA was achieved in 5 min at its *T_m_*_2_. Notably, the CD spectra recorded for g2CmeA at its corresponding *T_m_*_3_ continued changing for 30 min, indicating a slower unfolding rate. This result, along with the above data, indicates that *N*-linked glycans could play a pivotal intrinsic role in protein thermodynamic stabilization.

### Glycans modulate molecular assembly and protein-protein interaction.

**FIG 5:**
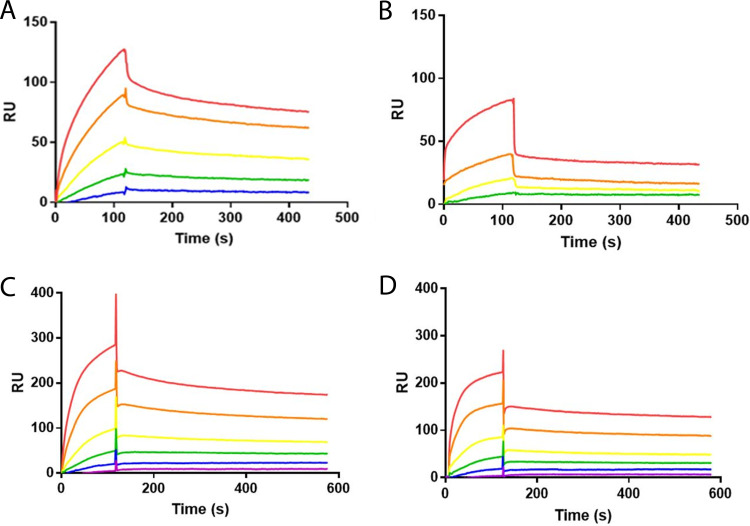
Glycosylation enhances interactions between CmeA variants and CmeC. (A and B) SPR analysis of CM5 chip with 900 RU of g2cmeA immobilized (A) and 1,040 RU of g0cmeA immobilized (B). Association of CmeC at pH 7.4 was assessed for 2 min, and dissociation was monitored for 5 min. The concentrations of CmeC were 2-fold dilutions from 2 × 10^−7^ M (red) to 1.25 × 10^−8^ M (blue) or 2.5 × 10^−8^ M (green). (C and D) SPR analysis of CM5 chip at pH 6.0 with 900 RU of g2CmeA immobilized (C) and 1,040 RU of g0CmeA immobilized (D). The association of CmeC was assessed for 2 min, and dissociation was monitored for 5 min. The concentrations of CmeC were 2-fold dilutions from 2 × 10^−7^ M (red) to 0.6 × 10^−8^ M (purple).

Unlike eukaryotes, there is no evidence that *N*-linked glycans modulate protein-protein interactions or complex assembly in prokaryotes. Surface plasmon resonance (SPR) was used to explore the potential role of the C. jejuni general *N-*linked glycans in modulating the interaction of glycoproteins with their cognate partners. We employed a CM5 chip with g0CmeA and g2CmeA immobilized through amine coupling. CmeC was then added to CmeA variant surfaces in different concentrations. In our model, g0CmeA and g2CmeA exhibited multiple interaction events with CmeC. These interaction events can be attributed to fast and slow association and dissociation rates. Quantitative analysis of the sensogram yielded convincing results for slow interactions; however, fast interactions could not be fitted in a model to generate accurate binding kinetics. More binding of CmeC was observed to the glycosylated CmeA, most of which followed slow on rate kinetics. At pH 7.4, both CmeA variants exhibited similar dissociation rate constants (*k*_off_) of 8.5 ± 1.0 × 10^–4^ s^–1^ for g0CmeA and 7.5 ± 0.6 × 10^–4^ s^–1^ for g2CmeA (Fig. 5A and B). Differences in the association rate constant (*k*_on_) were observed, g0CmeA *k*_on_ = 5.0 ± 0.8 × 10^4^ (M^–1^ s^–1^), while g2CmeA *k*_on_ = 1.5 ± 0.04 × 10^5^ (M^–1^ s^−1^). This difference in the *k*_on_ rate indicates that g2CmeA possess more binding pockets that allows slow yet high-affinity interactions with CmeC compared to g0CmeA. The dissociation constants (*K_d_*) derived from the binding kinetics analysis were 1.7 × 10^–8^ M and 5 × 10^–9^ M for g0CmeA and g2CmeA, respectively.

To investigate the effect of pH on binding kinetics, we measured CmeA-CmeC interactions at pH 6.0 (Fig. 5C and D). At this pH, CmeA-CmeC interactions were more avid, and a greater number of sites were bound. Similar to binding curves observed at pH 7.4, g2CmeA exhibited slower association and dissociation binding curves than g0CmeA. The number of sites for slow interaction were greater for g2CmeA, contributing to a modestly higher affinity for interaction with CmeC. In contrast, g0CmeA exhibited weaker affinity to CmeC. To confirm that variations in binding kinetics were not due to differences in structural orientation between g0CmeA and g2CmeA, both proteins were immobilized on nitrilotriacetic acid (NTA) chips using the C-terminal His_6_ tag; CmeC was then passed over the NTA chip at various concentrations. Binding kinetics indicated similar *k*_on_ and *k*_off_ values for both CmeA variants, although fewer sites were available (see Fig. S4 in the supplemental material). Interestingly, g2CmeA bound more CmeC than did g0CmeA, confirming the data seen with amine coupling. These results show a complex binding pattern between CmeA variants and CmeC. They also suggest an extrinsic role for *N-*linked glycans, exhibited in the variation in binding kinetics between g0CmeA and g2CmeA, where the glycosylated form of CmeA showed a greater proportion of higher-affinity interaction sites than did its nonglycosylated counterpart.

10.1128/mBio.02604-20.4 FIG S4 (a and b) 1,090 RU of immobilized g2CmeA (a) and 1,000 RU of immobilized g0CmeA (b). Binding to CmeC offered at 1 × 10^−7^ M (orange), 5 × 10^−8^ M (yellow), and 2.5 × 10^−8^ M (green) for 2 and 5 min of dissociation. CmeA variants were covalently associated by NHS/EDC after association through C-terminus His_6_ tag association with the Ni-NTA surface. Download FIG S4, JPG file, 0.02 MB. Copyright © 2020 Abouelhadid et al. 2020 Abouelhadid et al. This content is distributed under the terms of the Creative Commons Attribution 4.0 International license.

## DISCUSSION

Despite the scrutiny of general glycosylation pathways across the bacterial kingdom, little is still known about the direct role of bacterial glycans (31–34). It is widely accepted that mutations in general glycosylation pathways invariably affect virulence, colonization, adhesion, and motility (23, 31, 34–37). However, the mechanism by which impairment of glycosylation pathways reduce virulence has been long suggested to be due to pleotropic effects resulting from indirectly affecting global cellular pathways (31). Recent proteome-wide studies demonstrated that impairment of bacterial general glycosylation pathway led to an increase in chaperones and proteases involved in protein quality control, linking glycosylation to proteome stability (24, 38). Although unprecedented, these reports have not provided in-depth studies into the direct role that glycans exert on protein function. In C. jejuni, the regulation and mechanisms by which the major multidrug efflux pumps CmeABC extrudes antibiotics have been intensively investigated, but the role of posttranslational modification has been overlooked (16, 18, 26, 39–41).

A recent attempt to investigate the role of *N-*glycans in CmeABC provided interesting evidence that abrogation of CmeA glycosylation, solely, while CmeB and CmeC remained glycosylated, is sufficient to reduce C. jejuni chicken colonization substantially (41). However, this study by Dubb et al. was unable to assign a direct role for this posttranslational modification. This could be due to several reasons mainly related to the reconstruction of C. jejuni *N-*linked pathway, which was carried out in a *wecA*^+^ E. coli strain. WecA interferes with the biosynthesis of *N*-linked glycan by competing with the initiating transferase, PglC (undecaprenyl phosphate *N*,*N′*-diacetylbacillosamine 1-phosphate transferase). This event leads to heterogenous mixture of *N-*linked glycans that are built on the wrong reducing end sugar, GlcNAc, rather than diNAcBac (28, 29, 41, 42). Most importantly, unlike in C. jejuni, the authors reported a very low level of glycosylation of CmeA when CmeABC glycosylation was reconstructed in an *acrAB tolC* mutant E. coli strain, suggesting that such a model to study differences in multidrug efflux pump activity between glycosylated and nonglycosylated assemblies needs to be improved (41). Here, we demonstrate that disrupting glycosylation in CmeABC impaired its function. This impairment was confirmed using two independent assays: ethidium bromide accumulation, which assesses multidrug efflux kinetics in real time, and antibiotic MIC, which measures the endpoint of multidrug efflux pump activity to four structurally different antibiotics. Abrogation of CmeABC glycosylation resulted in a higher accumulation of ethidium bromide and a lowering antibiotic MIC in C. jejuni. This phenotype is not due to the low abundance of the CmeABC complex in a g0CmeABC strain. Immunoblot analysis did not show difference in CmeC expression in WTCmeABC and g0CmeABC from overnight cultures (Fig. 2C; see also Fig. S3 in the supplemental material). This aligns with previous findings demonstrating that CmeABC protein abundance was equal in C. jejuni and C. jejuni *pglB*::*aphA* (24, 38). Our results provide the first evidence that *N-*linked glycans are directly modulating the activity of CmeABC.

**FIG 6:**
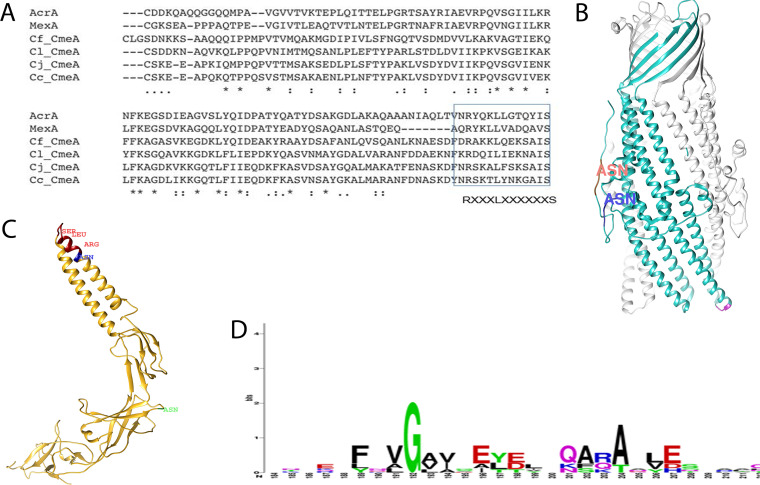
Analysis of binding sites in CmeA and CmeC. (A) Amino acid alignment of signal peptide processed CmeA orthologues. Conserved amino acids are denoted by an asterisk, similar amino acids are denoted by a colon, and weak amino acid similarity is denoted by a period. The amino acid sequences were retrieved from UniProt and aligned using Clustal Omega (52). The RLS attachment site is shown to be conserved among periplasmic accessory proteins from different strains. The localization of XRLS is highlighted in a blue box, showing the presence of ^123^N at X_–1_ in the conserved RLS motif in C. jejuni and C. coli (Cj_CmeA and Cc_CmeA, respectively) but not C. fetus or C. lari (Cf_CmeA and Cl_CmeA, respectively). (B) Structural representation focusing on chain A of the CmeC trimer (PDB 4MT4). Chain A is highlighted in cyan; ^32^N and ^49^N are highlighted in red and blue, respectively. The proposed attachment site VGA motif is highlighted in magenta, showing its distance from both glycosylation sites. (C) Structural prediction of CmeA. The signal-processed amino acid sequence was deposited in I-TASSER, and the best structural fit was based on the MexA model. The RLS motif is highlighted in dark red; ^123^N and ^273^N are highlighted in blue and light green, respectively, showing the close proximity of ^123^N to RLS motif in CmeA. (D) Analysis of outer membrane channels. CmeC, TolC, and OrpM show the conservation of Gly structurally located at the tip region of the coiled-coil α-hairpin domain among *Campylobacter* species, E. coli, and P. aeruginosa.

We carefully designed a strategy to achieve almost full glycosylation of CmeA glycosylated with the native C. jejuni *N-*linked glycans (Fig. 3A to E). This allowed us to study, with confidence, the biophysical properties of *N-*linked glycans. Bioinformatic studies investigating protein structural changes exerted by glycans have been inconclusive (43, 44). These studies rely on the *in silico* analysis of protein structure entries in the Protein Database Bank (PDB). While modern advances in crystallographic techniques pave the way for more structural studies, obtaining glycoprotein structure is still challenging and remains poorly represented in the PDB. Experimentally, our initial CD study of two CmeA variants consisting of glycosylated and nonglycosylated proteins showed that both have the same conformational fold; however, they confer subtle structural differences (Table 1). Small shifts have been observed in the percentages of α-helices and β-sheets between g0CmeA and g2CmeA of 2.2 and 0.8%, respectively. It is still unclear whether the structural variations are due to local stabilization resulting from the glycosidic bond between the asparagine side chain in the glycosylation site and *N-*linked glycans or global structural rearrangement due to the interaction of the glycan with other distant regions in the protein backbone. Biophysical studies on the intrinsic role of glycosylation demonstrated its direct effect on transforming the energy landscape of protein folding (45). The covalent attachment of glycans to asparagine could either perturb α-helices within an ordered structure or be well tolerated if located at the α-helix terminal (46). A previous survey on the structural assessment of glycosylation sites (SAGS) database reported that among 1,184 nonredundant occupied glycosylation sites, only 7% (88 sites) are located within ordered α-structures, whereby small residues (G, A, or S) are located adjacent or within *i* ± 3 and *i* ± 4 of the occupied site to compensate for steric effects due to the attachment of glycans (46). Interestingly, structural modeling of CmeA showed that ^123^N of DFNRS is located at the α-helical structure (Fig. 6). Protein alignment of CmeA sequences from 20 different *Campylobacter* species showed that only CmeA from C. jejuni and C. coli possesses a DXNRS glycosylation site (where X = Y or F). The glycosylation sequons are located at +4 positions to a conserved alanine. Notably, in C. jejuni and C. coli, a serine residue was found to be located at the −3 position relative to the glycosylation site (see Fig. S5A in the supplemental material). When we analyzed the second glycosylation site, DNNNS, we found that among the same 20 *Campylobacter* strains, 12 (60%) glycosylate the same location (see Fig. S5B in the supplemental material). Despite the glycovariants among *Campylobacter* species, diNAcBac seems to be conserved as the reducing end sugar in all of them; perhaps there is a specific role played by this monosaccharide at the different glycosylation sites.

10.1128/mBio.02604-20.5 FIG S5 Amino acid sequence alignment of *Campylobacter* species. CmeA amino acid sequences were retrieved from UniProt, aligned by CLUSTAL Omega. Conserved amino acids are denoted by asterisks. Two glycosylation sites are highlighted in red box. (a) DNFRS; (b) DNNNS. DFNRS is highlighted in a red box within the conserved arginine in the RLS motif; A and S are located at the X_–4_ and X_–3_ positions relative to the glycosylated N, respectively, while DNNNS in highlighted with the conserved F at X_–3_ and N at X_–1_ positions relative to the glycosylated N. Download FIG S5, JPG file, 0.1 MB. Copyright © 2020 Abouelhadid et al. 2020 Abouelhadid et al. This content is distributed under the terms of the Creative Commons Attribution 4.0 International license.

It has been suggested that *N-*linked glycans might enhance protein thermostability. Another C. jejuni glycoprotein, PEB3, was used to test the stabilization effect of *N-*linked glycans. The average melting temperature of PEB3 (K135E) variants were analyzed using SYPRO Orange ThermoFluor. Interestingly, the *T_m_* of glycosylated PEB3 was shown to be 4.7°C higher than its nonglycosylated counterpart, PEB3 (47). This aligns perfectly with the CD thermal melts of g0CmeA and g2CmeA. CD thermal melts showed that although both CmeA variants have the same apparent unfolding behavior, the *T_m_* of g2CmeA was 4.1°C ± 0.5 higher than that of g0CmeA. The transitional phases of both variants showed that g2CmeA seems to be responding to a rise in temperature via conformational rearrangements which were 2.3°C lower than g0CmeA (Fig. 4). CD spectra recorded after cooling showed that the structural rearrangements were reversible and the protein could fold again, suggesting that protein fold/unfolding *T_m_*_1_ for CmeA, *T_m_*_1_ and *T_m_*_2_ for g2CmeA are reversible. Remarkably, the unfolding behavior of g2CmeA at *T_m_*_3_ was different from that of g0CmeA at its corresponding *T_m_*_2_ in the conformational reversibility assay. The time taken to unfold g2CmeA was at least five times slower than g0CmeA, indicating a role played by *N-*linked glycans in conferring greater resistance to unfolding (Fig. 4D and E). We postulate that *N-*linked glycans could be stabilizing g2CmeA through a reduction in the unfolding rate in g2CmeA. This role that has been previously reported in eukaryotes whereby eukaryotic *N*-linked glycans stabilize hCD2ad through slowing of the unfolding rate of the protein (50 times slower) compared to its nonglycosylated counterpart (48).

Due to the lack of subcellular compartments, the extrinsic role of prokaryotic *N-*linked glycans in protein-protein interaction has not been fully appreciated. Despite the scarcity of glycoproteomic data, few molecular assemblies have been reported to have at least one of its components glycosylated (19, 49). We demonstrate a potential extrinsic role for *N-*linked glycans in the interaction between CmeA and CmeC. In an orthologous multidrug efflux pump, AcrAB-TolC, AcrA showed the presence of two populations of the same protein, interacting with different kinetics to TolC. The two populations (heterogenous ligand) contributed to a fast weak interaction and a slow strong interaction (Fig. 5A and B). The complexity of these interactions is exaggerated in C. jejuni due to the presence of *N-*linked glycans that could modulate the interaction of CmeA with CmeC. Quantitative analysis of the interaction kinetics of CmeA variants with CmeC showed that *N-*linked glycans correlated with an increase in the binding affinity to CmeC by 3.4-fold. That was demonstrated in the difference in *K_d_* between CmeA variants at pH 7.4. The difference in binding affinity was confirmed when CmeA variants were immobilized with the same orientation on a nickel chip. Recently, a pseudoatomic structure provided a detailed picture of interaction between AcrA and TolC. This elaborated the adaptor bridging-binding model that involved an intermesh cogwheel-like binding between AcrA and TolC (50). The conserved binding motif Val-Gly-Leu/Thr (VGL) is located at the tip region of the coiled coil α-hairpin of the protein, serving as a site of interaction with the RXXXLXXXXXXS (RLS) motif of AcrA (43). In light of this study, our computational analysis showed that CmeC from *Campylobacter* spp. does contain a truncated VGL motif, denoted VGA, while we found the RLS motif to be conserved among C. jejuni, C. lari, C. coli, and C. fetus (Fig. 6A). To understand whether *N-*linked glycans modulate protein-protein interaction, we analyzed the proximity of glycosylation sites to the VGA and RLS binding sites in both CmeC and CmeA, respectively. The glycosylation sites in CmeC were shown to be distant from the proposed binding site (Fig. 6B) and probably closer to the transmembrane domain of the protein. Interestingly, we found that one of the glycan-modified asparagines (^123^N) is at the X-1 position in the RLS motif and is conserved in C. jejuni and C. coli, but it is not in the tested 20 *Campylobacter* species (Fig. 6A and C; see also Fig. S5A). This strongly suggests that the localization of *N-*linked glycan adjacent to RLS might be affecting either the local site conformation and/or promote a stronger interaction with the VGA motif in CmeC, resulting in the interaction kinetics differences between g2CmeA and g0CmeA with CmeC observed by SPR in this study. In eukaryotes, it is established that *N*-linked glycans at different glycosylation sites in the same protein could play different roles. The roles of these *N-*linked glycans can be categorized as (i) promoting protein folding, (ii) modulating protein trafficking and localization, and (iii) affecting protein functionality (45). We hypothesize that *N-*linked glycans attached to different glycosylation sites in CmeA might play different role(s). Thus, *N-*linked glycans at ^123^N might be promoting interactions with CmeC, whereas glycosylation at ^273^N may promote protein stability.

Glycoproteomics relies heavily on two analytical platforms: targeting the released glycans and/or the enriching of glycoproteins (51). The lack of methods to release bacterial glycans and the scarcity of glycoprotein enrichment techniques limit in-depth mass spectrometry of bacterial glycoproteomes. This leads to an underappreciation of a common role played by bacterial glycans. In view of these limitations that hinders the study of bacterial glycoproteome, it is noteworthy that AcrB, an orthologue of C. jejuni glycoprotein CmeB, was reported to be glycosylated in the related *Epsilonproteobacteria* pathogen, Helicobacter pylori (49), whereas MdtA, an orthologue of C. jejuni glycoprotein CmeE of secondary multidrug efflux pump, was found to be also glycosylated in B. cenocepacia K56‐2 (31). Although H. pylori and B. cenocepacia possess *O-*glycosylation system(s), there is a potential similarity between the *O-*linked glycans and *N-*linked glycans in maintaining protein stability and/or mediating protein-protein interaction. We provide here the first detailed analysis of the role of posttranslational modification in mediating antimicrobial resistance. Our work demonstrates that *N-*linked glycans play a role in slowing the protein unfolding process and enhancing its thermostability, and they also modulate protein interaction with its cognate partner.

Our findings might indicate that a common evolutionary pressure led to the emergence of posttranslational modification of multidrug efflux pumps in pathogenic members of the *Epsilonproteobacteria* family. This evidence has been validated in a recent report showing that disruption of the *N-*glycosylation pathway in C. fetus affected the multidrug efflux pump activity in the bacterium, rendering a glycosylation-impaired variant more susceptible to antibiotics. Our study suggests a conserved role for oligosaccharyltransferase (OTase) attachment-dependent *N-*linked glycans, previously seen in eukaryotes, in expanding the functionality of the proteome repertoire across all domains of life. Our approach can be applied in general to interrogate prokaryotic general glycosylation systems. Our findings demonstrate that, regardless of glycan diversification among the domains of life, *N-*linked glycans seem to confer a common evolutionary intrinsic role.

## MATERIALS AND METHODS

### Bacterial strains and growth conditions.

Campylobacter jejuni 11168 and its derivatives—C. jejuni *cmeD*::*cat*, C. jejuni *cmeD*::*cat* wt*cmeABC*, and C. jejuni *cmeD*::*cat* g0*cmeABC*—were used in this study (Table 2). C. jejuni 11168H was grown on Columbia-based agar (CBA) or Muller-Hinton-based agar supplemented with 5% horse blood according to the manufacturer’s instructions. Strains were grown at 37°C in a variable atmospheric incubator (VAIN) cabinet (Don Whitely, UK) maintaining the following microaerophilic conditions: 85% nitrogen, 5% oxygen, and 10% carbon dioxide. All of the cloning experiments were performed in Escherichia coli DH10B (New England Biolabs). E. coli DH10B was used in the expression of CmeA and the cloning and expression of CmeC, whereas gCmeA was expressed in E. coli SBD1. E. coli strains were grown on either Luria-Bertani (LB) broth or LB agar, and antibiotics were added when necessary.

**TABLE 2:** Strains, plasmids, and primers used in this study

Strain, plasmid, or primer	Description	Source or reference
Strain		
E. coli DH10B	F^–^ *mcrA* Δ(*mrr*-*hsdRMS-mcrBC*) ϕ80d*lacZ*ΔM15 Δ*lacX74 endA1 recA1 deoR* Δ(*ara*, *leu*)*7697 araD139 galU galK nupG rpsL* λ^–^	New England Biolabs, UK
E. coli SDB1	F^–^ λ^–^ IN(*rrnD-rrnE*)*1 rph-1* Δ*waal* Δ*wecA*	30
C. jejuni 11168H	Hypermotile variant of C. jejuni 11168	21
C. jejuni 11168H *cmeD*::*cat*	C. jejuni 11168H *cmeD* is inactivated by chloramphenicol cassette insertion.	This study
C. jejuni *cmeD*::*cat* wt*cmeABC*	C. jejuni 11168H *cmeD*::*cat*, *cmeC* is His_6_ tagged, followed by kanamycin and *cj0364*, to allow selection on a CBA plate supplemented with antibiotic and homologous recombination, respectively.	This study
C. jejuni *cmeD*::*cat* g0*cmeABC*	C. jejuni 11168H *cmeD*::*cat*, *cmeABC* is glycosylation deficient by altering N→Q in C. jejuni glycosylation sequon (D/E-X-N-X-S/T, where X is any amino acid other than proline); *cmeC* is His_6_ tagged, followed by kanamycin and *cj0364*, to allow selection on a CBA plate supplemented with antibiotic and homologous recombination, respectively.	This study
		
Plasmids		
pEXT21Cj*pglB*	*pglB* cloned in pEXT21 under *lac* promoter	T. Scott et al., unpublished data
pWA2	Soluble periplasmic His_6_-tagged CmeA under the Tet promoter in pBR322	32
pMH5	Soluble periplasmic His_6_-tagged CmeA under the Tet promoter in pCAYC184	32
pACYC(*pgl*)	C. jejuni heptasaccharide coding sequence under the Tet promoter in pCAYC184	32
pJMK30	*aphA* gene cloned in BamHI restriction site	42
pAT3	Membrane-bound 10×His-tagged CmeC driven to periplasm by DsbA signal peptide under the l-arabinose promoter in pEC145	This study
pATN	*cmeD*::*cat* cloned in pJET1.2	This study
pMH3	*cmeABC* locus cloned in pJET1.2	This study
pMHT	*aphA* cloned in BamHI site in pMH3	This study
pMHTF	*cj0364* cloned in SacII site in pMHT	This study
pATM	g0*cmeABC* locus cloned in pJET1.2	This study
pATMN	*aphA* cloned in BamHI site in pATM	This study
pATKH	*cj0364* cloned in SacII site in pATMN	This study
		
Primers		
FWDCmeA	AGCGAAGTTAAAGAAATTGGAGCAC	
REVCmeC	TTTT*CCGCGG*ATT*GGATCC*CATTATGATGATGATGAT GATGATGTTCTCTAAAGACATATCT	
FWDcj0364	TTTT*CCGCGG*ATTCTCTAAATAAATTAAAAATCTTTGTCT	
REVcj0364	TTTT*CCGCGG*CATTGAACCTTTTTGGAGGGATTTTTCC	
CmeCFwd1	TTTT*GCTAGC*GCCGCCCCAAATTTAAATATTCCCGAAGCAAACTATAGCATTG	
CmeCRev1	TTTTT*GTCGAC*CTAATGATGATGATGATGATGATGATGATGATGTTCTCTAAAAGACATATCTAAATTTTTTGATTC	

### Inactivation of *cmeD* and generation of C. jejuni *cmeD*::*cat*, *cmeD*::*cat* wt*cmeABC*, and *cmeD*::*cat* g0*cmeABC*.

The nucleotide sequence of *cmeD* gene was commercially synthesized (Clontech) to also carry a chloramphenicol-resistant gene; *cat* was inserted in the middle of *cmeD* to disrupt the gene. The DNA was then released by restriction digestion with EcoRV and cloned in pJET1.2, according to manufacturer’s instructions, to generate pATN. Cloning of *cmeABC-aphA* was achieved by the following methods. The *cmeABC* locus was amplified using the primers FWDCmeA and REVCmeC with Phusion polymerase (New England Biolabs, UK) with C. jejuni 11168H genomic DNA as a template, and the His_6_ tag was added at the C terminus of the CmeC to track its expression. The PCR amplicon was cloned in pJET1.2 according to the manufacturer’s instructions to give pMH3, which was was then cut by BamHI to introduce the kanamycin-resistant gene *aphA*, to be used as an antibiotic selection marker after homologous recombination in C. jejuni 11168H to generate pMHT. To add homologous recombination arms for *cmeABC*-*aphA*, pMH3 was cut by SacII to ligate *cj0364* at the 3′ end of *aphA* to generate pMHTF. For g0*cmeABC-aphA*, each asparagine in the noncanonical glycosylation sequon (D/E-X_1_-N-X_2_-S/T, where X_1_ and X_2_ represent any amino acid except proline) was altered to glutamine *in silico*, and the nucleotide sequence of g0*cmeABC* was synthesized by (Clontech). DNA was then treated as described above to generate pATKH.

To generate C. jejuni *cmeD*::*cat*, electroporation of pATN into C. jejuni 11168H was carried out as previously described (10). The transformants were selected on CBA plates supplemented with 10 μg/ml chloramphenicol, and the double-crossover event was confirmed by PCR; this strain was then used as a parent strain to generate other mutants. Plasmids pMHT and pATK were electroporated into C. jejuni *cmeD*::*cat* to generate C. jejuni *cmeD*::*cat cmeC*::*cmeC-aphA* and C. jejuni *cmeD*::*cat cmeABC*::*cmeABC-*(N→Q)-*aphA*, respectively. Transformants were selected on CBA plates supplemented with 10 μg/ml chloramphenicol and 30 μg/ml kanamycin, and the double-crossover event was confirmed by PCR.

### Antibiotic sensitivity test (Etest).

*C. jejuni* 11168H was grown in suspension in Mueller-Hinton broth equivalent to a 1.0 MacFarland standard, and 100-μl aliquots were spread plated on dry Mueller-Hinton agar plates supplemented with 5% sheep blood (Oxoid, UK). The plates were then left for 5 to 10 min to dry before an antibiotic strip (Oxoid) was added. Plates were incubated at 37°C overnight. The MIC was read directly from the strip at the point where the zone of inhibition of bacterial growth intersected with the antibiotic concentration on the strip.

### Ethidium bromide accumulation assay.

Bacterial cells were grown to mid-log phase (optical density at 600 nm [OD_600_] = 0.4 to 0.5). Cells were harvested, washed, and resuspended in 0.1 M sodium phosphate buffer (pH 7; previously incubated in the VAIN) to an OD_600_ of 0.2. The cells were then incubated in the VAIN apparatus for 15 min at 37°C before a 100-μl aliquot was withdrawn to indicate time zero. Ethidium bromide (Sigma, UK) was added to final concentration of 2 μg/ml, and fluorescence was measured at 530-nm excitation and 600-nm emission using an M3 plate reader (Molecular Devices).

### Expression of CmeA and gCmeA.

Protein expression was assessed in E. coli strains unless stated otherwise. Nonglycosylated CmeA, g0CmeA, was expressed in E. coli DH10B carrying pMH5 plasmid; gCmeA was expressed in E. coli SDB1 carrying pWA2 and pACYC(*pgl*); and g2CmeA was expressed in E. coli SDB1 carrying pEXT21Cj*pglB* pWA2, and pACYC(*pgl*). Initiating cultures were grown overnight in LB broth supplemented with appropriate antibiotics at 37°C under shaking condition. The following day, 10 ml of culture was withdrawn from the shake flask to inoculate 400 ml of LB broth supplemented with appropriate antibiotics. To achieve optimal glycosylation of CmeA, PglB was expressed from pEXT21Cj*pglB* by the addition of 0.5 mM ITPG at an OD_600_ of 0.5 to 0.6. Cultures were incubated at 37°C for 24 h with shaking. Cultures were centrifuged, and cell pellets were washed with binding buffer (300 mM NaCl and 50 mM NaH_2_PO_4_ with 25 mM imidazole) and passed twice through a high-pressure cell homogenizer (Stanstead Works, UK). Cell debris was removed by centrifugation at 20,000 × *g* for 45 min. The supernatant was collected, followed by incubation with 0.2 ml of Ni-NTA for 1 h at 4°C, and then washed with 50 ml of binding buffer and eluted four times in 0.5 ml of elution buffer (300 mM NaCl and 50 mM NaH_2_PO_4_ with 250 mM imidazole).

### Cloning and expression of CmeC.

To express CmeC in E. coli, *cmeC* lacking signal peptide sequence was amplified by PCR with CmeCFwd1 and CmeCRev1 using C. jejuni 11168H genomic DNA as a template. The amplicon was then cut by NheI and SalI and cloned into pEC415 downstream of the DsbA signal peptide sequence to give pAT3. E. coli carrying pAT3 was grown in LB media supplemented with ampicillin (100 μg/ml) overnight at 37°C under shaking conditions. On the following day, 10 ml was withdrawn from the overnight culture to inoculate 400 ml of LB media. Cells were grown to an OD_600_ of 0.5 to 0.6, and 0.2% l-arabinose was added to induce the expression of CmeC. Cultures were incubated at 37°C for 24 h with shaking at 180 rpm. The cultures were centrifuged, and the cell pellets were washed with binding buffer (300 mM NaCl and 50 mM NaH_2_PO_4_ with 25 mM imidazole) and passed twice through cell homogenizer (Stanstead Works). Cells debris was removed by centrifugation at 20,000 × *g* for 45 min and then collected and incubated in binding buffer with 2% n-dodecyl-β-d-maltoside (DDM) for 3 h at 4°C. The mixture was then centrifuged at 15,000 × *g* for 10 min. The supernatant was collected, diluted with binding buffer, and incubated with 0.2 ml of Ni-NTA for 1 h at 4°C. The sample was then washed with 50 ml of binding buffer and eluted four times in 0.5 ml of elution buffer (300 mM NaCl and 50 mM NaH_2_PO_4_ with 250 mM imidazole).

### Mass spectroscopy.

In-gel reduction, alkylation, and digestion with trypsin or chymotrypsin was performed on the gel sample prior to subsequent analysis by mass spectrometry. Cysteine residues were reduced with dithiothreitol and derivatized by treatment with iodoacetamide to form stable carbamidomethyl derivatives. Trypsin digestion was carried out overnight at room temperature after initial incubation at 37°C for 2 h. The peptide sample was resuspended in 30 μl of resuspension buffer (2% acetonitrile in 0.05% formic acid), 10 μl of which was injected to be analyzed by liquid chromatography-MS/MS. Chromatographic separation was performed using a U3000 UHPLC NanoLC system (Thermo Fisher Scientific, UK). Peptides were resolved by reversed-phase chromatography on a 75-μm C_18_ column (50-cm length) using a three-step linear gradient of 80% acetonitrile in 0.1% formic acid. The gradient was delivered to elute the peptides at a flow rate of 250 nl/min over 60 min. The eluate was ionized by electrospray ionization using an Orbitrap Fusion Lumos (Thermo Fisher Scientific) operating under Xcalibur v4.1.5. The instrument was first programmed to acquire using a Universal_CID method by defining a 3-s cycle time between a full MS scan and MS/MS fragmentation. This method takes advantage of multiple analyzers on Orbitrap Fusion Lumos and drives the system to use all available parallelizable time, resulting in decreasing the dependence on method parameters (such as DDA). The instrument was programmed to acquire in automated data-dependent switching mode, selecting precursor ions based on their intensity for sequencing by collision-induced fragmentation using a TopN CID method. The MS/MS analyses were conducted using collision energy profiles that were chosen based on the mass/charge ratio (*m/z*) and the charge state of the peptide.

### CD spectroscopy.

All CD spectra of gCmeA and CmeA were acquired in 0.5-mm rectangular cell pathlength using a Chirascan spectrometer (Applied Biophysics, UK) equipped with a Quantum NorthWest TC125 Peltier unit. Temperature-dependent confirmation changes were monitored at wavelengths of 260 to 195 nm for gCmeA (0.2 mg/ml) and CmeA (0.2 mg/ml) in 10 mM sodium phosphate–75 mM sodium chloride–10% glycerol buffer (pH 8.0) during a stepwise increase in temperature from 6 to 94°C. Temperatures were measured directly with a thermocouple probe in the sample solution. Melting temperatures were determined from the derivative CD-temperature spectra and fitted using a Levenberg-Marquardt algorithm (LMA) on the van’t Hoff isochore (Global 3 [Global Analysis for T-Ramp, version 1.2 build 1786]; Applied Photophysics Ltd., 2007-2012). For the conformation reversibility study, far-UV CD spectra were recorded at 20°C, increased to the *T_m_*, and recooled to 20°C. The temperature at each elevated *T_m_* was kept constant for 5 min, and the CD spectrum was recorded to assess the rate of protein unfolding process.

### Surface plasmon resonance.

For coupling of CmeA and gCmeA to the CM5 sensor chip, carboxyl groups on the surface were activated by injecting a 1:1 mixture of 0.4 M 1-ethyl-3-(3-dimethylaminopropyl)-carbodiimide (EDC) and 0.1 M *N*-hydroxysuccinimide (NHS) for 7 min at 5 μl/min. CmeA and gCmeA were diluted to 10 to 20 μg/ml in 0.1 M acetate (pH 5.5) and immobilized at 5 μl/min. Immobilization was stopped when the required number of response units (RU) was achieved. This was followed by injecting 1 M ethanolamine (pH 8.5; 7 min at 5 μl/min) to inactivate excess reactive groups. To account for nonspecific binding, a control flow cell was generated using the same method described minus the protein immobilization step. To couple CmeA and gCmeA to a NTA chip, the chip was cleaned and loaded with NiCl_2_ (0.5 mM). The flow cells were then activated as described above, and CmeA and gCmeA (10 μg/ml in HBSP buffer [10 mM HEPES, pH 7.4, 0.005% {wt/vol}, and Surfactant P20 {GE Healthcare}]) were loaded into appropriate flow cells until appropriate RU levels were achieved. Subsequently, the flow cells were treated with ethanolamine as described above to block remaining activated sites.

CmeC at various concentrations (3 nM to 0.2 μM) was analyzed at a constant temperature of 25°C under continuous flow of HBS-PE buffer (10 mM HEPES [pH 7.4], 3 mM EDTA, 0.005% [wt/vol] surfactant P20 [GE Healthcare]) at 30 μl/min (sufficient to prevent mass transfer effects) at pH 7.4 for 3 min of association and a dissociation time of 5 min. Experiments at pH 6.0 were performed using 10 mM MES (pH 6.0), 3 mM EDTA, and 0.005% (wt/vol) surfactant P20 (GE Healthcare) The surface chip was regenerated by injecting 0.1 M triethanolamine (pH 11.5). Data were analyzed using BIAevaluation software (v4.1.1; Biacore; GE Healthcare, Amersham). Blank flow cell controls were subtracted. The *K_d_* was defined between 10 s after the end of the sample injection and 300 s later.
