# Supplementary figures and images for: Characterization of Posttranslationally Modified Multidrug Efflux Pumps Reveals an Unexpected Link between Glycosylation and Antimicrobial Resistance
Source: mBio. 2020 Nov 17;11(6):e02604-20. doi: 10.1128/mBio.02604-20 (PMC7683400; doi:10.1128/mBio.02604-20)

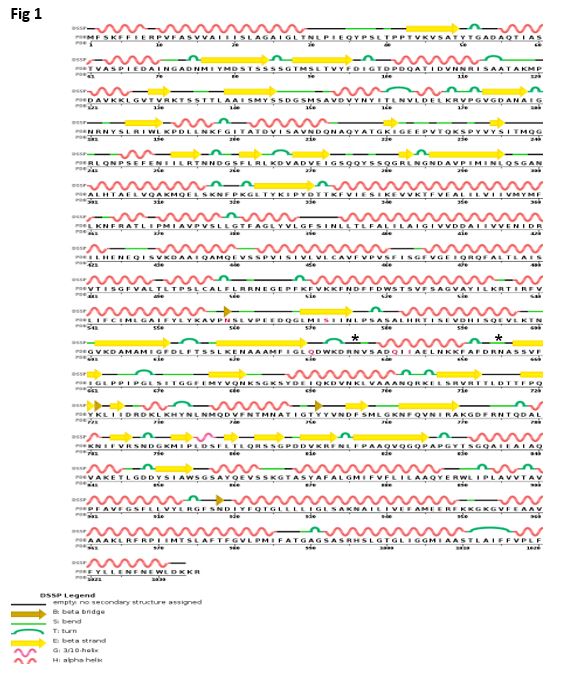

Supplement: FIG S1 [file mBio.02604-20-sf001.jpg]

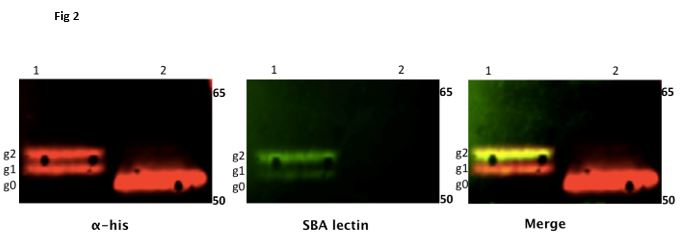

Supplement: FIG S2 [file mBio.02604-20-sf002.jpg]

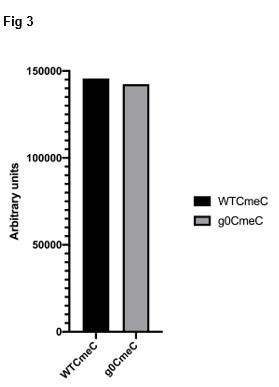

Supplement: FIG S3 [file mBio.02604-20-sf003.jpg]

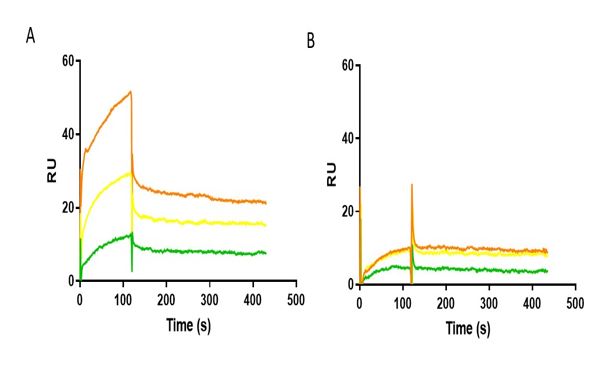

Supplement: FIG S4 [file mBio.02604-20-sf004.jpg]

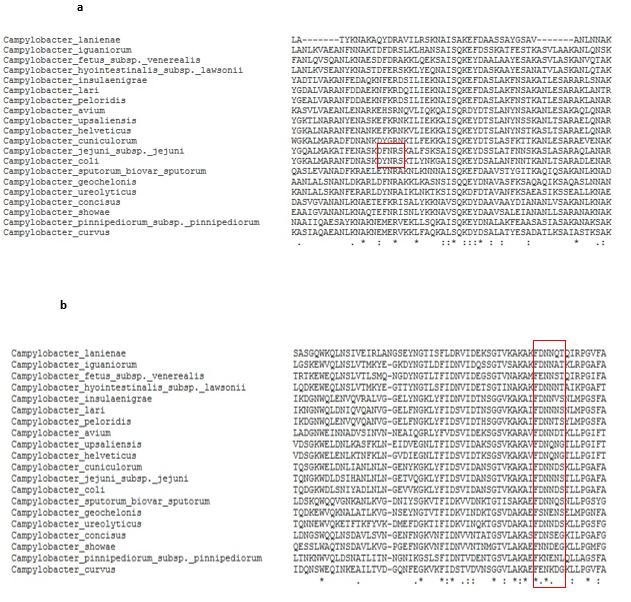

Supplement: FIG S5 [file mBio.02604-20-sf005.jpg]
